# Effects of Different Types of Calcium Sulfate Hydrates Incorporated into Dental Calcium Silicate Cement on Hardening, Flow, Anti-Washout, and Biological Properties

**DOI:** 10.3390/ma19143014

**Published:** 2026-07-13

**Authors:** Yun-Jeong Park, Hyeon Seo, Weon-Young Choi, Umugire Alphonse, Ho-Jun Song, Yeong-Joon Park

**Affiliations:** Department of Dental Materials, School of Dentistry, Chonnam National University, Gwangju 61186, Republic of Korea; 197560@jnu.ac.kr (Y.-J.P.); swapoo@naver.com (H.S.); onong0209@jnu.ac.kr (W.-Y.C.); aumugire@jnu.ac.kr (U.A.); songhj@jnu.ac.kr (H.-J.S.)

**Keywords:** calcium silicate cement, calcium sulfate, setting time, washout resistance

## Abstract

Calcium sulfate (CaSO_4_; CS) is added to dental calcium silicate cement (CSC) to regulate the hydration speed of the tricalcium aluminate phase. However, most commercial CSC products do not specify the CS hydration type, and reports on the role of CS are scarce. Therefore, the effects of the hydration type of the CS compound incorporated into CSC were evaluated. The synthesized CSC clinker was confirmed using XRD, TEM, and FT-IR. Subsequently, a ZrO_2_ (5 μm) radiopacifier was added at 20 wt%, and different types of CS compounds (either anhydrite, hemihydrate, or dihydrate) were added at 5 wt%. The hydration type of CS significantly affected the setting time and flowability (*p* < 0.05). The CS hemihydrate addition group showed the highest flowability, according to the ISO and LVDT methods (*p* < 0.05). The CS dihydrate addition group showed the lowest flowability, fastest setting time, and highest washout resistance and compressive strength. The 5 wt% addition of CaSO_4_ anhydrite significantly increased the setting time (*p* < 0.05). The CS-containing CSC groups showed cell viability levels similar to those of the CSC control group. These results provide valuable information for optimizing the selection of CS hydration type for manufacturing CSC tailored to specific clinical situations.

## 1. Introduction

Dental calcium silicate cement (CSC), commonly referred to as mineral trioxide aggregate (MTA) in the dental field, is a root canal treatment material based on Portland cement (PC). Due to its excellent characteristics, including high biocompatibility, antimicrobial properties, and a superior sealing ability [1,2,3,4,5], CSC is used for various clinical purposes in endodontics for a broad range of clinical procedures, including pulp capping, pulpotomy, apexification, root perforation repairs, intra/extra-root resorption, canal sealing, orthograde root filling, and retrograde root-end filling [6,7].

The main crystalline phases of CSC primarily consist of tricalcium silicate (C_3_S), dicalcium silicate (C_2_S), and tricalcium aluminate (C_3_A) [8,9]. Among them, C_3_A, which is known for its rapid hydration rate, contributes to a phenomenon called “flash set”, which adversely affects the workability and initial strength [10,11]. Therefore, the method of adding about 3–5 wt% of calcium sulfate (CaSO_4_; CS) to Portland cement has been used for a long time to prevent the rapid hydration of C_3_A [12,13,14].

CS has also been used in the medical and dental fields as a bone substitute material and as a barrier membrane for guided bone regeneration [15]. CS offers advantages in clinical applications, including its lack of a proinflammatory response; its complete resorption after transplantation, creating a calcium-rich environment at the transplantation site; and the ability to combine the pharmacologic agents and osteogenic growth factors prior to its placement, effectively releasing it into wound sites, which is favorable for tissue regeneration [16,17,18].

CS exists in different forms of crystalline phases depending on its hydration state. Calcium sulfate anhydrite (CaSO_4_) is known to exist in three forms: Type I, which exists at temperatures above 1180 °C; Type II, which is insoluble; and Type III (γ-CaSO_4_), which is soluble. Calcium sulfate hemihydrate (CaSO_4_·½H_2_O) occurs in two forms, α and β, depending on its crystal size, surface area, and lattice defects [19,20]. Lastly, calcium sulfate dihydrate (CaSO_4_·2H_2_O) exists as a stable crystalline hydrate phase under ambient conditions. Among these, calcium sulfate dihydrate (CaSO_4_·2H_2_O) and some anhydrite (orthorhombic CaSO_4_) are known to occur naturally [21,22].

In the field of industrial Portland cement, it has been reported that the hydration rate of C_3_A is influenced by the added sulfate type [23,24]. However, most manufacturers of current dental CSC products do not specify clearly which hydration form of CS is contained, and research on the effects of the CS hydration type is very limited [25].

Therefore, CSC clinkers were synthesized, and three CSC formulations containing different hydration forms of CS were prepared. It was hypothesized that the hydration form of CS would influence the properties of the CSCs. Accordingly, this study aimed to investigate how different hydration forms of CS affect the physical properties and biocompatibility of CSCs.

## 2. Materials and Methods

### 2.1. Materials

CaCO_3_, SiO_2_, Al_2_O_3_, MgO, K_2_CO_3_, Na_2_CO_3_, CaSO_4_ anhydrite (orthorhombic), CaSO_4_ hemihydrate, CaSO_4_ dihydrate, and ZrO_2_ were acquired from Sigma-Aldrich (St. Louis, MO, USA). Isopropanol and phosphate-buffered saline (PBS) were obtained from Merck (Darmstadt, Germany) and Gibco (Carlsbad, CA, USA), respectively.

#### Manufacture of Calcium Silicate Cement

Three types of CSC clinkers were manufactured with lime saturation factors (LSF) of 88 (CSC #1), 98 (CSC #2), and 108 (CSC #3). The CaCO_3_, SiO_2_, Al_2_O_3_, MgO, K_2_CO_3_, and Na_2_CO_3_ powders were mixed with isopropanol according to the respective wt% composition of each group using a ball mill with 3 and 5 mm zirconia balls for 6 h. The resulting mixture was dried at 80 °C for 24 h, after which the dried powder was combined with water and shaped into pellets. The pellets were subsequently dried at 80 °C for an additional 24 h. Thermal treatment was then conducted from room temperature to 600 °C at a rate of 5 °C/min in a superkanthal electric furnace and maintained at 600 °C for 30 min. The temperature was subsequently raised to 1450 °C, and the clinkers were sintered for 15 min. The sintered clinkers were cooled to 1250 °C, and then rapidly quenched to room temperature in a water-cooled port using a cooling fan [24]. The sintered clinkers of CSC #2 were pulverized using an alumina mortar and pestle, and subsequently milled using a vibration mill and a jet mill to obtain fine powder (mean particle size of 3.31 μm, D90 = 4.92 μm) for sample preparation and property evaluation. CS anhydrite, CS hemihydrate, and CS dihydrate were each added to the CSC clinker powder at 5 wt%. Additionally, 20 wt% of ZrO_2_ was incorporated as a radiopacifying agent.

### 2.2. Characterization of the Synthesized Calcium Silicate Cement Clinker Powder

#### 2.2.1. X-Ray Diffraction Analysis of Crystalline Phases of Clinkers

The crystalline phases of finely pulverized powders of the clinkers (obtained using a jet mill) were characterized using X-ray diffractometry (XRD; X’Pert PRO multi-purpose diffractometer, PANalytical, Almelo, The Netherlands). Diffraction data were collected using Cu Kα radiation operated at 40 kV and 30 mA. Scans were performed over a 2θ range of 5–75° with a step size of 0.026° and a scanning speed of 2°/min. A quantitative phase analysis was performed with the Rietveld refinement method using the X’Pert HighScore Plus software (PANalytical, The Netherlands).

#### 2.2.2. Transmission Electron Microscopy

The crystal structure of the clinker powder was analyzed using a transmission electron microscope (TEM, Tecnai F20 UT, Thermo Fisher Scientific, Waltham, MA, USA). The powder was dispersed in an alcohol solution and then attached to a Cu grid to prepare the specimens for TEM observation.

#### 2.2.3. Fourier-Transform Infrared Spectroscopy Analysis

The chemical bonding of the clinker powder was analyzed using a Fourier-transform infrared spectrophotometer (Hyperion 2000, Bruker, MA, USA) equipped with an attenuated total reflectance accessory. Spectra were collected in the range of 4000–600 cm^−1^ with a resolution of 4 cm^−1^, and 64 scans per sample.

#### 2.2.4. Particle Size Analysis

The particle size distribution was determined using a laser diffraction particle size analyzer (MasterSizer 2000, Malvern Instruments Ltd., Worcestershire, UK). Isopropyl alcohol was employed as the dispersing medium, and measurements were conducted three times to obtain the average value.

### 2.3. Preparation of the Sample

The CSC powders, each prepared by adding 5 wt% of CS (anhydrite, hemihydrate, or dihydrate), were mixed with distilled water at a water/powder ratio (W/P) of 0.6 for 1 min under controlled conditions (23 ± 1 °C and 50 ± 5% relative humidity). CSC without a CS addition was used in the control group for comparison.

### 2.4. Flowability

#### 2.4.1. ISO Method

**Figure 1:**
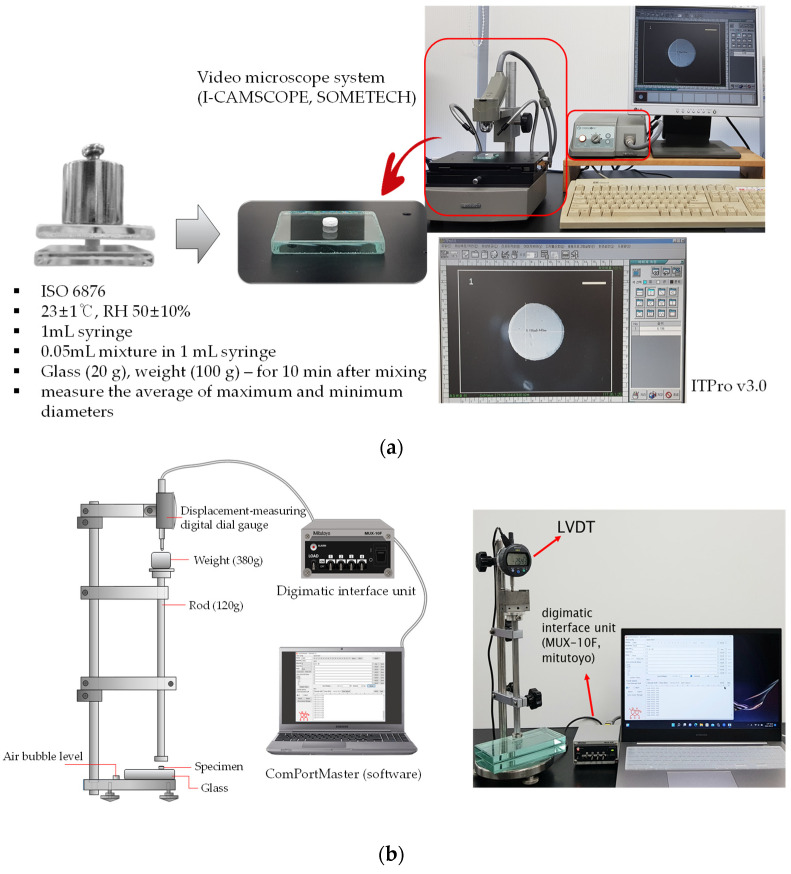
Instrumental set-up for flow test: (**a**) ISO method [26]; (**b**) LVDT method, which consisted of load-displacement-measuring equipment, including an LVDT (linear variable displacement transducer) digital dial gauge, a loading device with a weight of 500 g (including the rod), a digimatic interface unit, and the ComPort Master software. The mixed sample was placed between two glass plates.

The flowability of the samples was assessed in accordance with ISO standard 6876 [26]. After filling 0.05 mL of the mixture into a 1mL syringe, it was dispensed onto a glass plate. After 3 min, a 40 mm × 40 mm glass plate (approximately 20 g) was then carefully positioned on top, followed immediately by the application of an additional weight of 100 g on top of the glass plate. After an elapsed time of 10 min, the maximum and minimum diameters of the spread specimen were measured using an image analyzer (I-Camscope, Sometech Co., Ltd., Seoul, Republic of Korea). The results were obtained by using the average of the largest and smallest diameters (Figure 1a). The experiment was repeated seven times.

#### 2.4.2. LVDT Method

Figure 1b is a schematic diagram of the linear variable displacement transformer (LVDT) measuring system, which was used for the evaluation of flowability. An amount of 0.05 mL of the mixture was filled into a 1 mL syringe. After 60 s of mixing, the mixture was dispensed onto a glass plate, and a cover glass was placed on top. After 90 s had elapsed since mixing, a load of 500 g was applied. The displacement was measured until 10 min after mixing. The measurement values were continuously digitized by a digital interface data logger connected to the LVDT of the digital gauge and recorded using the ComPortMaster software (Withrobot Inc., Seoul, Republic of Korea). The percentage shortening was calculated based on how much the height of the specimen decreased compared with the initial measurement. The experiment was repeated seven times.

### 2.5. Setting Time Evaluation

The setting time was determined following ISO standard 6876 in a chamber with controlled environmental conditions (23 ± 1 °C and 60 ± 5% relative humidity). The freshly mixed cement was poured into a metal mold (10 mm in diameter and 2 mm in depth), and the surface was leveled to make the surface flat. It was then allowed to cure. The setting time was measured by gently placing a Gilmore needle (2 ± 0.1 mm tip diameter, 100 ± 0.5 g mass) onto the material until the indentation mark was no longer visible. The elapsed time was recorded. The experiment was repeated seven times.

### 2.6. Washout Resistance

The mixture was poured into a metal mold (10 mm internal diameter, 2 mm depth). The metal mold was secured to the center of a polisher’s rotating plate using a bisected fixture (spinning at 300 rpm). After 150 s from the start of mixing, water was sprayed for 5 s at a flow rate of 60 mL/min through an 18 G needle positioned 45 mm from the specimen surface. The washed-out image was scanned using a 3D scanner (T710, Medit, Seoul, Republic of Korea) to generate a stereolithography (STL) file. The STL data were subsequently converted to a standard for the exchange of product model data (STEP) format using the open-source FreeCAD software (version 0.20, available at https://www.freecad.org). The volume of the washed-out area was calculated using Computer-Aided Three-Dimensional Interactive Application version 5 (Catia V5) program (Dassault Systèmes Korea Corp., Incheon, Republic of Korea) [24]. The experiment was repeated five times.

### 2.7. Compressive Strength

The compressive strength test was conducted in reference to ISO standard 9917-1. The mold used for sample preparation was a split-ring mold with a specimen-contacting part made of Teflon; it had three holes (6.0 ± 0.1 mm in height and 4.0 ± 0.1 mm in diameter). The mixed CSC material was placed into the mold using an amalgam carrier, applying slight pressure, and excess material was leveled flat using a spatula. The filled mold was transferred to the storage chamber maintained at 37 ± 1 °C and 100% relative humidity, and allowed to cure for 3 h. Subsequently, the specimens were gently polished using 400-grit SiC paper and then removed from the mold to inspect them for bubbles or damaged areas. Each set of seven specimens was stored in the chamber for 1, 3, and 7 days prior to testing.

The compressive strength measurement was conducted using a universal testing machine (Instron 4302, Instron Co., High Wycombe, UK). The specimen was placed and compressed in the longitudinal direction at a crosshead speed of 1.0 mm/min.

### 2.8. Penetration Resistance

The resistance at a penetration depth of 0.8 mm was measured over time using a push–pull gauge fitted with a 2 mm diameter cylindrical indenter tip. After filling the aluminum mold (W: 28 mm, D: 5 mm, H: 2 mm) with freshly mixed CSC paste, the penetration resistance was measured at 3, 6, 9, 12, 15, 20, 30, 45, and 60 min after the start of mixing under controlled conditions (23 ± 1 °C and 60–70% relative humidity). The LabView program was used to set the penetration tip to descend to a depth of 8 mm into the CSC sample at a speed of 30 µm/s and to read data 20 times per second. The measured resistance force was converted to megapascals (MPa) by dividing it by the penetrating tip’s cross-sectional area. The experiment was repeated five times. Subsequently, the results were plotted as a spline curve graph. The penetration resistance values over time were extracted from the image graph using the Engauge digitizer program, an open-source digitizing tool developed by Mark Mitchell [24]. The average elapsed time (min) required to attain penetration resistance values of 50 and 60 MPa was calculated.

### 2.9. Biocompatibility

#### 2.9.1. Cell Culture

L-929 mouse fibroblast-like cells (NCTC clone 929, Korean Cell Line Bank, Korean Cell Line Research Foundation, Seoul, Republic of Korea) were seeded into 96-well plates (1 × 10^4^ cells/well) and incubated for 24 ± 1 h at 37 ± 1 °C under 5% CO_2_. The RPMI complete medium used for the cell culture was prepared by supplementing Dulbecco’s minimum essential medium (DMEM; Gibco, #11995) with 5% fetal bovine serum (FBS; Gibco, #16140) and 1% penicillin/streptomycin (Gibco, #15140).

#### 2.9.2. Sample Elution

The CSC was mixed with a water-to-powder ratio of 0.6 and placed into Teflon molds (diameter: 10 mm, height: 2 mm). The mixture was cured at 37 ± 1 °C and 100% relative humidity for 24 h. Subsequently, the cured CSC specimens were removed from the Teflon molds and sterilized by exposing both sides to UV light for 30 min each. The sterilized CSC specimens were then placed in 1 mL of DMEM and eluted at 37 °C in an incubator for 24 h. After elution, the supernatant was centrifuged and filtered through a 0.22 μm syringe filter (Jet Biofil, Guangzhou, China) to remove particulates.

#### 2.9.3. MTT Assay

The L-929 cells were cultured in a 96-well plate at a density of 1 × 10^4^ cells per well for 24 h at 37 °C in a 5% CO_2_ incubator. After incubation, the prepared eluate was diluted to concentrations of 100%, 50%, and 25%, and 100 μL of each dilution was added to the wells. The plate was then incubated for an additional 24 h at 37 °C in the incubator. Wells with cells replaced with DMEM without eluate served as the control.

For the assessment of cell viability, the eluate was removed, and the cells were washed with phosphate-buffered saline (PBS; Gibco, #10010). Then, 100 μL of an MTT solution (3-(4,5-dimethylthiazol-2-yl)-2,5-diphenyltetrazolium bromide, Sigma-Aldrich, #M2128) at a concentration of 0.5 mg/mL was added to each well, and the plate was incubated for 4 h at 37 °C. After removing the MTT solution, 100 μL of dimethyl sulfoxide (DMSO; Sigma-Aldrich) was added to dissolve the formed formazan crystals, and the plate was stored at room temperature for 15 min. The absorbance was then measured at 570 nm using a microplate reader (Sunrise, Tecan, Grödig, Austria). The cell viability was normalized to the control and expressed as a percentage. The test was replicated five times.

### 2.10. Statistical Analysis

The results for the flowability, setting time, washout resistance, and penetration resistance were analyzed using the Kruskal–Wallis test followed by Dunn’s post hoc multiple comparison test. The compressive strength and MTT assay results were analyzed using a two-way analysis of variance (ANOVA), followed by Tukey’s post hoc test for multiple comparisons. The correlation between the flowability results obtained using the ISO and LVDT test methods was analyzed using a Pearson and Spearman correlation analysis. SPSS version 26.0 (IBM Corp., Armonk, NY, USA) was employed for all operations, with the level of significance set at *p* < 0.05.

## 3. Results

### 3.1. Characterization of the Synthesized Calcium Silicate Cement Clinker Powder

#### 3.1.1. X-Ray Diffraction Analysis

**Figure 2:**
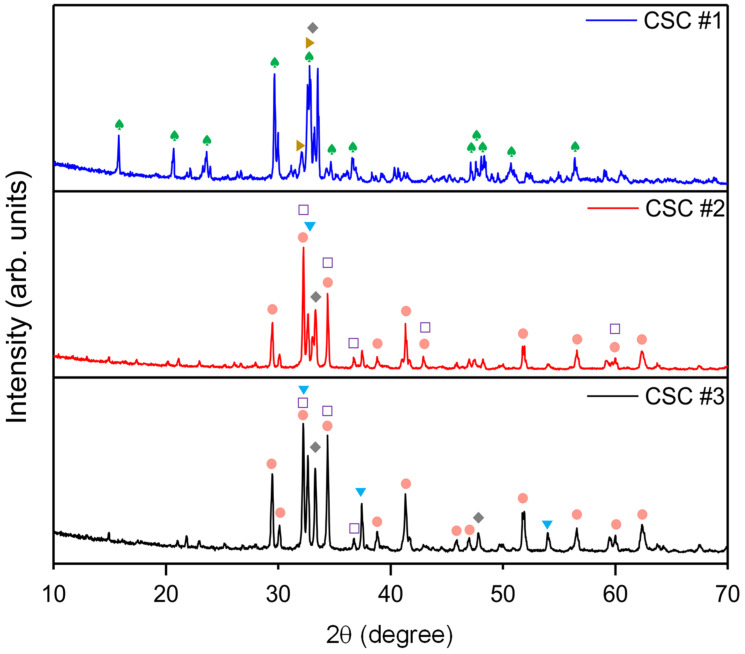
X-ray diffraction (XRD) patterns of calcium silicate cement (CSC) clinker powder. **●**: tricalcium silicate, □: dicalcium silicate, ♠: γ-dicalcium silicate, **▶:** β-dicalcium silicate, **◆**: tricalcium aluminate, ▼: calcium oxide.

The X-ray diffraction patterns of the three experimental calcium silicate cement clinker powders are shown in Figure 2, and the crystalline phase composition obtained from Rietveld refinement is summarized in Table 1. Three types of CSC clinkers with lime saturation factor (LSF) values of 88 (CSC #1), 98 (CSC #2), and 108 (CSC #3) were tested. CSC #1 showed no presence of tricalcium silicate (C_3_S) and had 53.4% γ-dicalcium silicate (γ-C_2_S). CSC #3 exhibited the highest proportion of C_3_S, at 67.4%, and calcium oxide (CaO), at 6.8%, while the calcium carbonate (CaCO_3_) proportion was at 0.7%. CSC #2 exhibited 53.4% C_3_S, 16.8% β-dicalcium silicate (β-C_2_S), and 27.9% tricalcium aluminate (C_3_A) (Figure 2, Table 1). Hence, CSC #2 was deemed the most suitable composition and used for the property evaluation experiments.

#### 3.1.2. Particle Size

**Figure 3:**
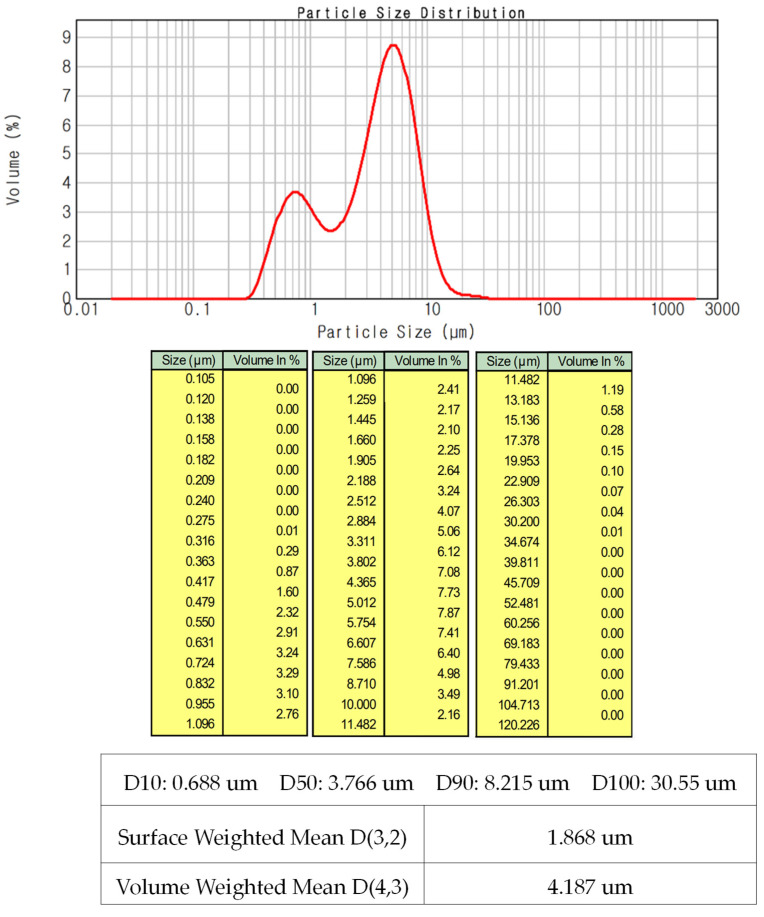
Particle size distribution of synthesized calcium silicate cement powder after vibration and jet milling.

The average particle size of the synthesized CS cement powder after vibration and jet milling was 3.31 µm, with D_10_ and D_90_ values of 0.688 and 8.215 μm, respectively (Figure 3).

#### 3.1.3. Fourier-Transform Infrared Spectroscopy Analysis

**Figure 4:**
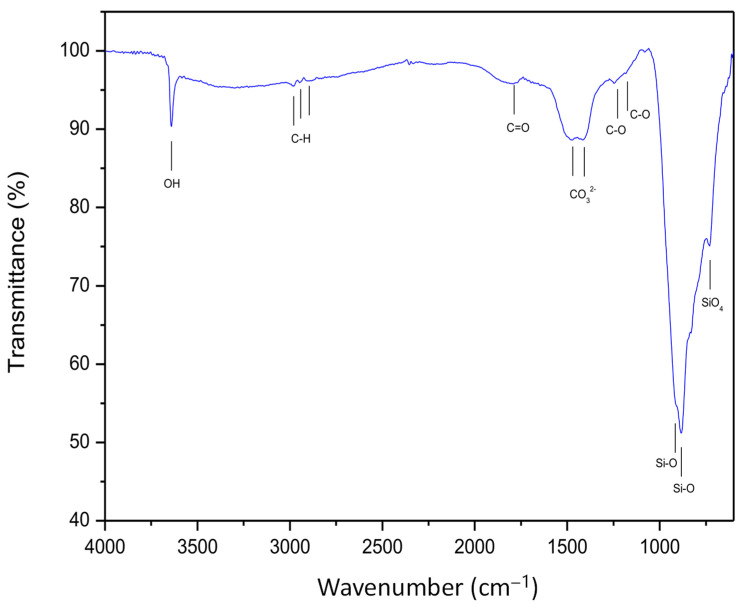
FT-IR spectrum analysis of CSC clinker powder finely pulverized using a jet mill.

The chemical bonding characteristics of finely pulverized CSC clinker powder was analyzed using FT-IR spectrum shown in Figure 4. Peaks corresponding to Si-O stretching vibration were observed at 883 and 912 cm^−1^, while peaks corresponding to CO_3_^2−^ were observed at 1415 and 1473 cm^−1^. A peak corresponding to O-H stretching vibration was detected at 3640 cm^−1^.

#### 3.1.4. Transmission Electron Microscopy Observation

**Figure 5:**
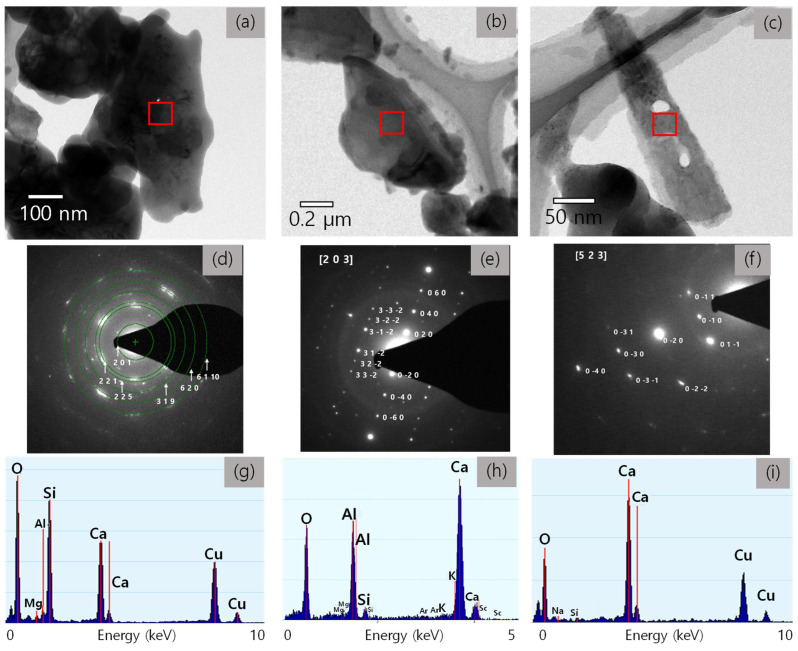
Transmission electron microscopy (TEM), selected area electron diffraction (SAED), and energy dispersive spectroscopy (EDS) analyses of the CSC clinker powders. (**a**–**c**) TEM images of three representative particles. Insets indicate the regions where the SAED and EDS analyses were performed. (**d**–**f**) Corresponding SAED patterns obtained from the red box areas of (**a**–**c**), respectively. (**g**–**i**) Corresponding EDS spectra obtained from the red box areas of (**a**–**c**), respectively. The red vertical lines indicate the position of the characteristic X-ray peaks for the corresponding elements. The particles were identified as monoclinic tricalcium silicate (**a**,**d**,**g**), cubic tricalcium aluminate (**b**,**e**,**h**), and tetragonal calcium oxide (**c**,**f**,**i**).

Transmission electron microscopy (TEM), selected area electron diffraction (SAED), and energy-dispersive spectroscopy (EDS) analyses were performed to identify the crystalline phases of the synthesized SCS clinker powders (Figure 5). Three representative particles were selected for the analysis (Figure 5a–c), and the boxed regions indicate the areas where the SAED and EDS measurements were conducted.

For the particle shown in Figure 5a, the corresponding SAED pattern (Figure 5d) exhibited ring-type diffraction patterns, indicating a polycrystalline structure caused by the relatively thick specimen. The measured diffraction rings were indexed using the JCPDF card (#42-0551) and found to be consistent with monoclinic tricalcium silicate (Ca_3_SiO_5_, lattice parameters: a = 12.233 Å, b = 7.034 Å, c = 24.960 Å, α = 90.0°, β = 90.1°, and γ = 90.0°). The EDS spectrum (Figure 5g) showed strong Ca, Si, and O peaks, confirming the composition of tricalcium silicate.

For the particle shown in Figure 5b, the SAED pattern (Figure 5e) exhibited discrete diffraction spots. The diffraction pattern was indexed along the [2 0 3] zone axis and matched well with the simple cubic structure of tricalcium aluminate (3CaO⋅Al_2_O_3_, lattice parameters: a = b = c = 15.269 Å and α = β = γ = 90°). The EDS spectrum (Figure 5h) revealed dominant Ca, Al, and O peaks. A weak Si peak was also observed, suggesting that the partial substitution of aluminum by silicon may have occurred in the tricalcium aluminate structure.

For the particle shown in Figure 5c, the SAED pattern (Figure 5f) was indexed as tetragonal calcium oxide (CaO, lattice parameters: a = b = 3.540 Å, c = 5.910 Å, and α = β = γ = 90°). The diffraction spots corresponded well with the [5 2 3] zone axis. The EDS spectrum (Figure 5i) showed only Ca and O peaks, confirming the calcium oxide composition. The Cu peaks observed in the spectrum originated from the TEM grid used for specimen preparation.

### 3.2. Flowability

**Figure 6:**
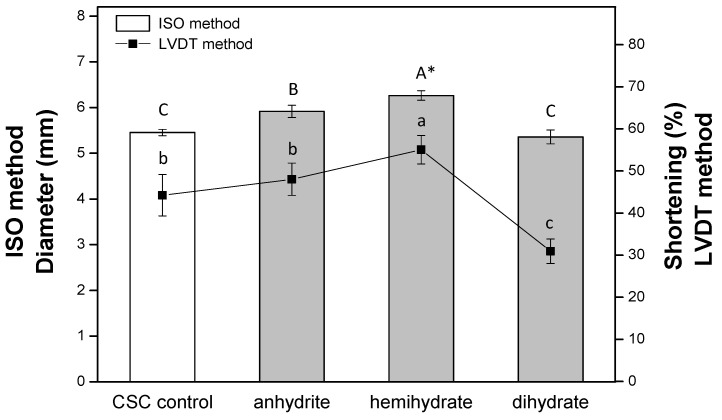
The results of the flowability test as determined using the International Organization for Standardization (ISO) method and the linear variable displacement transformer (LVDT) method. * Different uppercase letters (within the ISO method) and different lowercase letters (within the LVDT method) indicate significant differences between the groups at *p* = 0.05, with *n* = 7 replicates for each group.

The flowability was tested using the ISO method and the LVDT-based method for CSC containing different types of CS hydrates. Figure 6 shows that the CS hemihydrate addition group exhibited the highest flowability, with diameter values of 6.26 ± 0.10 mm in the ISO flow test and percentage shortening values of 55.05 ± 3.38% in the LVDT-based method. On the other hand, the CS dihydrate addition group showed the lowest flowability, with corresponding values of 5.36 ± 0.15 mm and 30.93 ± 2.92%, respectively. The flowability of the groups was in the following order: hemihydrate, anhydrite, control, and dihydrate groups. The hydration state of calcium sulfate markedly influenced the flow characteristics of the cement (*p* < 0.05). In Figure 6, significant differences between the groups are indicated by different uppercase letters for the ISO flow test and different lowercase letters for the LVDT-based method. The flowability measured using either the ISO or LVDT method showed consistent results for each CS hydrate type (ρ = 0.83, *p* < 0.001).

### 3.3. Setting Time

**Figure 7:**
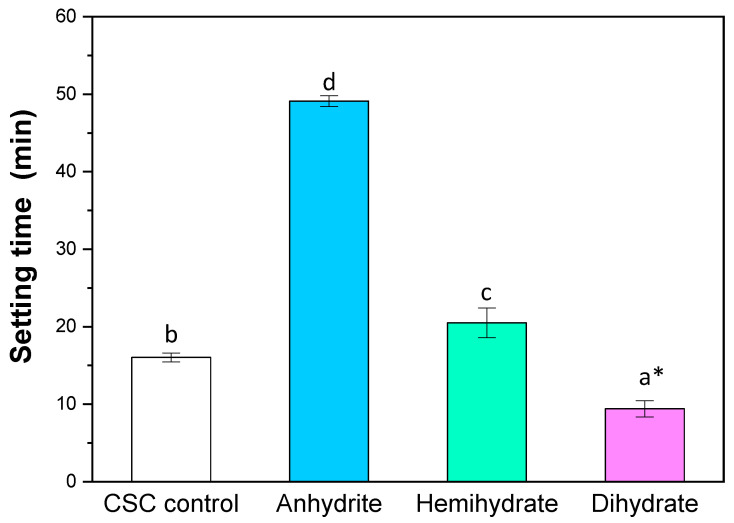
Setting time. * Different small letters indicate significant differences between the groups at *p* = 0.05, with *n* = 7 replicates for each group.

Figure 7 shows that the setting time of the CS dihydrate addition group was the shortest, at 9.75 ± 0.77 min, while compared with the CSC control group, the setting times of the CS anhydrite addition group and the CS hemihydrate addition group were longer, at 49.11 ± 0.68 and 20.50 ± 1.91 min, respectively.

### 3.4. Washout Resistance

**Figure 8:**
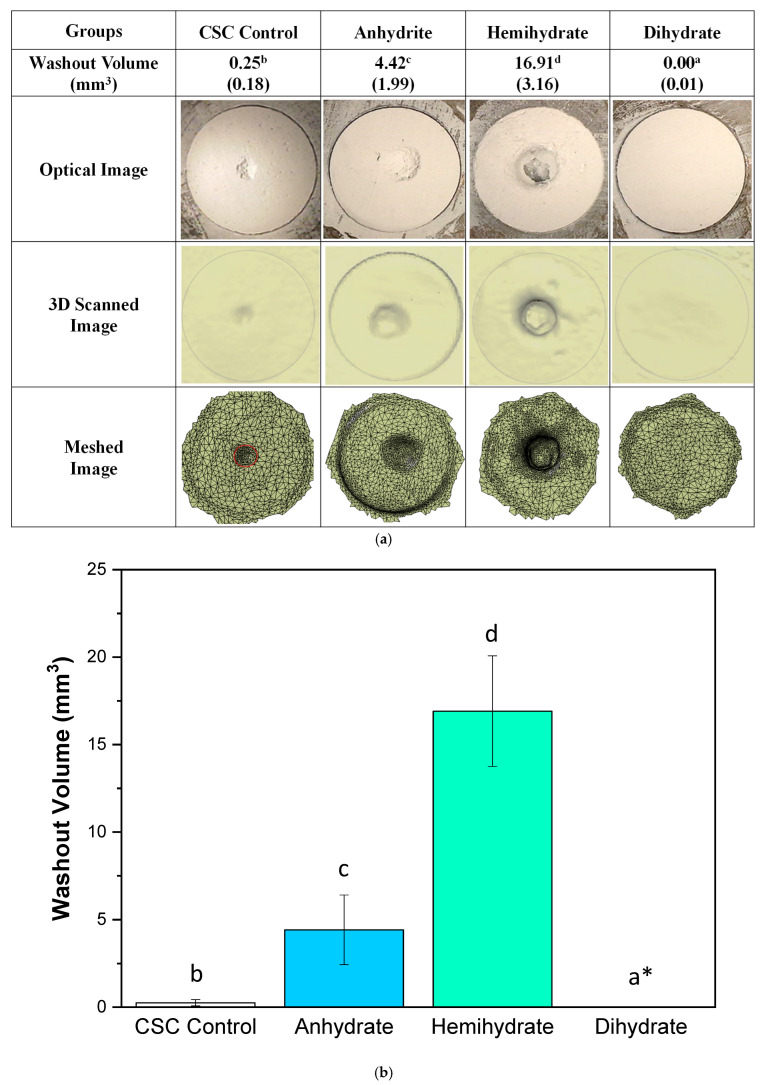
(**a**) Washout volume (mm^3^) for different groups, presented alongside optical images, 3D scanned images, and Catia transformation images (*n* = 5). Different small letters indicate significant differences between groups at the *p* = 0.05 level. The washout volume was calculated using Catia V5 software. (**b**) Washout volume (mm^3^). * Different small letters indicate significant differences between groups at *p* = 0.05, with *n* = 5 replicates for each group.

Figure 8a,b shows that the CS dihydrate addition group exhibited the lowest washout volume at 0.00 ± 0.01 mm^3^, while the CS hemihydrate addition group, which showed the highest flowability, demonstrated the lowest washout resistance at 16.91 ± 3.16 mm^3^ (*p* < 0.05).

### 3.5. Compressive Strength

**Figure 9:**
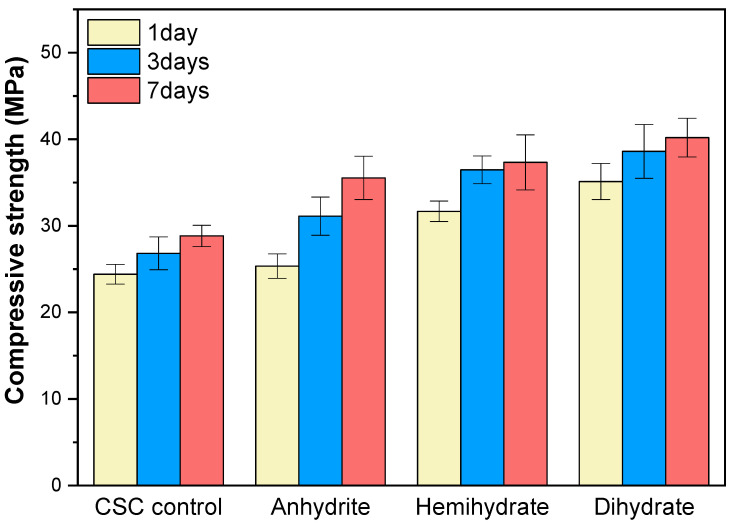
Compressive strength (MPa) at 1, 3, and 7 days, with *n* = 7 replicates for each group.

The compressive strength increased over time (*p* < 0.05). Compared with the CSC control and CS anhydrite addition groups, the CS hemihydrate and CS dihydrate addition groups exhibited a higher compressive strength at 1 and 3 days (Table 2, Figure 9).

### 3.6. Penetration Resistance

Table 3 shows the average elapsed time (min) until the penetration resistance reached specific penetration resistance values against indenter penetration. The elapsed time to reach 50 and 60 MPa of the penetration resistance decreased in the following order: anhydrite group > hemihydrate group > CSC control group > dehydrate group.

**Figure 10:**
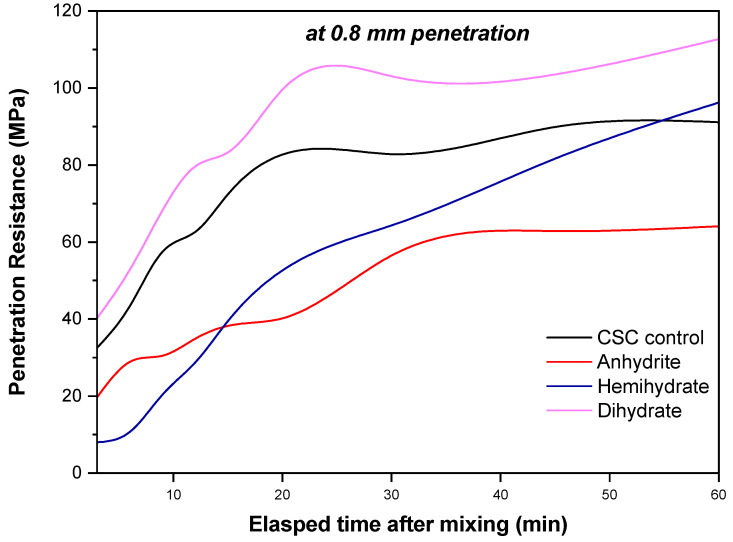
Spline curves of the average resistance values (MPa) of mixed calcium silicate cement specimens against 0.8 mm penetration as a function of elapsed time after mixing.

Figure 10 presents the spline curves of the average resistance values of mixed CSC specimens against 0.8 mm penetration, plotted over time after mixing.

### 3.7. Biocompatibility

**Figure 11:**
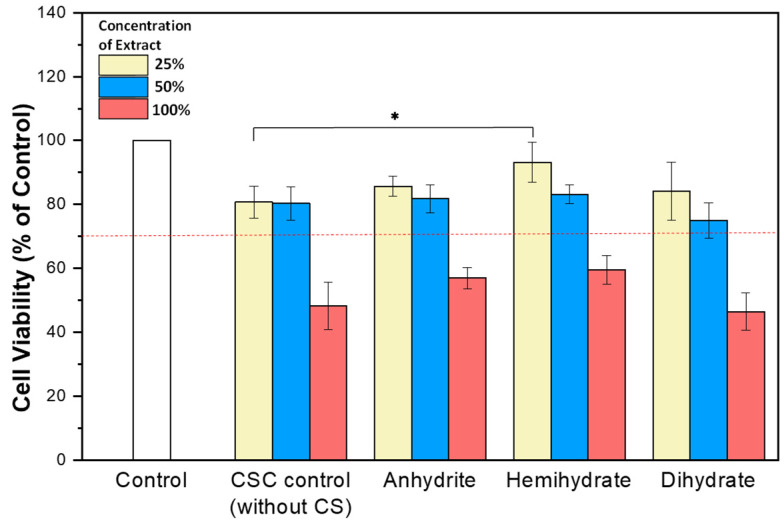
Cell viability (CV; % viability relative to the control group) of calcium silicate cement according to the various hydration types of the incorporated calcium sulfate. The CV at the 25% and 50% extract dilutions satisfied the ISO requirement of above 70% (marked with the red dotted line). * For the 25% extract dilution groups, the CS-hemihydrate-containing CSC had a significantly higher CV compared with the CSC control group (CS-non-containing) at *p* = 0.05, with *n* = 5 replicates for each group.

The cell viability remained above 75% compared with the control when the leachate was diluted to 50% and 25%, suggesting minimal cytotoxicity (Figure 11). However, the undiluted leachate at 100% exhibited a slight cytotoxicity, lower than 60%, compared with the control without CS. The experimental groups with a CS addition showed cytotoxicity levels similar to those of the CSC control (without CS) for the 100% leachate (*p* < 0.05).

## 4. Discussion

In this study, three types of calcium sulfate (CS) with different hydration forms were added to experimental calcium silicate cement (CSC) to evaluate the effects of the different hydration forms on the physical properties and biocompatibility of the cement. In this study, three CSC clinker compositions with different lime saturation factor values (LSF = 88, 98, and 108) were evaluated to determine the most suitable phase assemblage for subsequent investigation.

In Figure 2 and Table 1, the XRD refraction patterns and relative proportions of the crystalline phases of the experimentally synthesized calcium silicate cement clinker powders are shown, respectively.

The CSC #1 group, with an LSF value of 88, exhibited significant dusting during cooling from the sintering temperature. This dusting phenomenon can be linked to the presence of a γ-dicalcium silicate (γ-C_2_S) phase, as identified from the XRD refraction pattern of the CSC #1 group (Figure 2). The relative proportion of γ-C_2_S in the CSC #1 group was 53.4% (Table 1). The dusting phenomenon could be attributed to the transformation of β-C_2_S to γ-C_2_S around the temperature range from 650 to 700 °C during cooling [14,27,28,29]. This transformation is associated with a volume expansion of approximately 14%, leading to the mechanical disintegration of the clinker into fine powder, thus producing self-pulverized dusts. Importantly, γ-C_2_S is considered hydraulically inactive or poorly reactive compared with β-C_2_S, resulting in a limited contribution to hydration and strength development [27,30,31]. Therefore, the high fraction of γ-C_2_S in CSC #1 renders it unsuitable for use as a hydraulic dental cement, and the CSC #1 composition was removed as a candidate for further physical property evaluation experiments.

In contrast, CSC #3 (LSF: 108) contained the highest proportion of C_3_S (67.4 wt%) but also exhibited the presence of free calcium oxide (CaO) in the highest proportion among the tested groups. The presence of excess CaO (6.8 wt%) indicates an incomplete clinker reaction and may adversely affect the dimensional stability due to delayed hydration and expansion, which is undesirable for clinical applications [9,14,29,32]. Free CaO can rapidly hydrate to form calcium hydroxide, increasing the alkalinity; however, uncontrolled hydration may compromise the structural integrity and long-term stability of the cement matrix.

Among the tested compositions, CSC #2 (LSF 98) showed a balanced phase composition, consisting primarily of C_3_S, β-C_2_S, and tricalcium aluminate (C_3_A), with minimal free CaO (Figure 2, Table 1). This phase assemblage is considered optimal for the hydraulic performance, as C_3_S governs early strength development and calcium ion release, while β-C_2_S contributes to long-term hydration and stability [32,33]. Additionally, C_3_A plays a critical role in early hydration reactions and interacts with sulfate ions to form ettringite, which influences the setting behavior and nucleation processes [34,35]. Therefore, CSC #2 was selected for this study as it provides a suitable balance between the reactivity, phase stability, and hydration performance. This optimized clinker composition is used to establish a reliable baseline for investigating the effect of the CS compound on the rheological, mechanical, and biological properties of calcium-silicate-based dental cements. The synthesized CSC #2 clinkers were pulverized to fine powders with an average particle size of 3.31 μm (Figure 3) using a vibration mill followed by a jet mill for characterization of the synthesized CSC and to make the mixed CSC samples for the property evaluation.

The FTIR analysis of the CSC #2 clinker powder revealed characteristic bands associated with anhydrous calcium silicate phases (Figure 4). The prominent absorption bands at 883 cm^−1^ and 912 cm^−1^ were attributed to Si–O stretching vibrations, and the absorption band around 730 cm^−1^ was attributed to bending vibrations of silicate tetrahedra, particularly Si–O–Si and/or Si–O–Ca linkages in calcium silicate phases, which confirmed the presence of crystalline calcium silicate phases (C_3_S and C_2_S) [36,37,38]. The peaks at 1415 and 1473 cm^−1^ corresponded to the asymmetric stretching vibrations of CO_3_^2−^ groups, suggesting the presence of CaCO_3_ [39,40]. The sharp absorption band at approximately 3640 cm^−1^ was assigned to the O–H stretching vibration of Ca(OH)_2_, likely formed by the hydration of residual free CaO upon exposure to atmospheric moisture [41,42].

Together with the XRD and FT-IR analyses, the TEM/SAED/EDS analyses (Figure 5) confirmed that the synthesized CSC #2 clinker consisted predominantly of tricalcium silicate (C_3_S), tricalcium aluminate (C_3_A), dicalcium silicate (C_2_S), and calcium oxide (CaO), which are the principal crystalline phases of Portland-cement-based dental materials. C_3_S and C_2_S are crucial crystal phases in the hydration reaction. The C_3_S phase is known for its early strength among CSC crystal phases, influencing early setting, while the C_2_S phase is known for its slow hydration and involvement in long-term strength [43,44]. The presence of C_3_S (Figure 5a,d,g) and C_3_A (Figure 5b,e,h), together with CaO (Figure 5c,f,i), demonstrated that the applied synthesis conditions successfully produced a typical hydraulic cement clinker system, consistent with previous reports on MTA composition [7,32,45].

From a functional perspective, these phases play distinct and complementary roles in hydration and setting behavior. C_3_S is the primary phase responsible for early strength development, as its rapid hydration leads to the formation of calcium silicate hydrate (C–S–H) and calcium hydroxide, which establish the initial mechanical framework of the cement; this process subsequently contributes to long-term strength, bioactivity, and sustained calcium ion release [29,46,47].

The nanostructured nature of C–S–H contributes to the formation of a dense, mechanically stable matrix. In contrast, C_3_A exhibits a high reactivity and rapidly participates in early hydration reactions, significantly influencing the initial setting and handling properties. The presence of CaO further contributes to alkalinity and calcium ion availability, which are critical for biological responses and mineralization in endodontic applications [34,45,46,48].

Importantly, the identification of these phases provides a mechanistic basis for interpreting the effects of CS incorporation that were investigated in this study. The interaction between C_3_A and sulfate species plays a critical role in regulating early hydration, as the addition of CS promotes the formation of ettringite [Ca_6_Al_2_(SO_4_)_3_(OH)_12_·26H_2_O], which precipitates around the surface of cement particles rather than as uncontrolled needle-like C_3_A hydration products [14,29]. This sulfate-controlled ettringite layer moderates the rapid reaction of C_3_A, prevents the premature loss of flowability, and ensures a more controlled setting process during the early stages of hydration [29,32].

Flowability was evaluated using two different testing methods. Previously, flowability tests for CS cement were performed in accordance with ISO 6876 (Figure 1a) [26,49]. However, there are some shortcomings in that experimental procedure. The conventional ISO-based method provides only a static endpoint measurement and has limitations in capturing continuous flow behavior during cement manipulation. In particular, it is challenging to align the glass plate accurately in the center of the mixed material since it needs to be performed by eye, making it difficult to find the exact center. This inaccuracy would potentially affect the measurement reproducibility.

To address these limitations, this study adopted a new flowability evaluation method using a linear variable displacement transducer (LVDT) (Figure 1b). An LVDT is a type of transducer used to measure linear distance, and its digitized results make it easy to obtain continuous measurements of the displacement during material spreading, offering a significant advantage [50,51].

The results demonstrated that the hydration form of the CS included in the CSC composition significantly influenced the cement’s flow behavior.

In Figure 6, the flowability test results using the LVDT and ISO methods show that the CS hemihydrate addition group exhibited the highest flowability, while the CS dihydrate addition group showed the lowest flowability. These differences are conjectured to be attributed to differences in the dissolution behavior of the different hydration types of the CS component, all of which can affect the paste consistency and rheological changes over time after mixing the cement with water [52,53].

Notably, the results obtained from the ISO method and the LVDT method showed a similar trend, suggesting that the new LVDT method is a viable alternative to the ISO method. There was a statistically significant positive correlation between the LVDT-based measurement results and the ISO flow test results (r = 0.81, *p* < 0.001). Although the two methods are based on different measurement principles and are therefore not directly interchangeable in absolute numerical terms, their comparable trends indicate that the LVDT-based method is a valid and reliable supplementary approach for evaluating CSC flowability.

Furthermore, the LVDT method offers several practical advantages over the ISO flow test method, including continuous flow monitoring, an improved measurement sensitivity, and a greater adaptability to customized experimental setups, supporting its applicability as a replaceable method for assessing the flow behavior of calcium-silicate-based cements.

The incorporation of CS compounds significantly influenced the flowability and setting time of CSC (Figure 6 and Figure 7), which can be explained by a dissolution–precipitation mechanism governed by solubility and nucleation effects.

In the presence of CS, the reaction of rapidly hydrating C_3_A is dramatically modulated. Initially, ettringite (Ca_6_Al_2_(SO_4_)_3_(OH)_12_·26H_2_O) crystals develop on the C_3_A particles as the main hydrate phase forms, which hinders the rapid hydration reaction of C_3_A. When the added CS has all been consumed in the CSC, the rate of the C_3_A hydration reaction rapidly increases again. The delay in C_3_A hydration depends on the quantity of the CS in the system [29,54].

Figure 7 shows that the setting time was shortest in the CS dihydrate group, followed by the CSC control, CS hemihydrate, and CS anhydrite groups. Cement with a longer setting time is more susceptible to dissolution during a root canal treatment, while cement with an extremely short setting time can pose technical challenges during clinical handling [55]. Therefore, it is essential to ensure an appropriate setting time based on the specific endodontic treatment procedure being performed.

In this study, the shorter setting time of the hemihydrate compared with the anhydrite group was attributed to the higher solubility of CS hemihydrate than that of CS anhydrite, which promotes rapid dissolution and generates a highly supersaturated solution for the CS dihydrate crystal formation. This result can be attributed to the relative solubility differences of the CS phases, which follow the order of CS hemihydrate (~0.9 g/100 mL) > CS anhydrite (~0.3 g/100 mL) > CS dihydrate (~0.2 g/100 mL) [56,57]. The higher solubility of CS hemihydrates accelerates ion release and enhances supersaturation, which acts as a driving force for the rapid nucleation and growth of CS dihydrate crystals, leading to the formation of an interlocking crystalline network responsible for early setting [58,59]. In contrast, the lower solubility of CS anhydrite delays ion release and supersaturation development, due to which the nucleation needed to initiate the heterogeneous precipitation of solids is delayed, resulting in a prolonged setting time (Figure 7) [54,60].

Consistently, the flowability results shown in Figure 6 demonstrate that the hemihydrate group exhibited the highest flowability, which can be attributed to the increased ion release and improved lubrication within the paste, facilitating particle mobility. This enhanced workability is directly linked to the higher dissolution rate of this group and agrees with previous findings that CS hemihydrate improves the rheological behavior while accelerating hydration [61]. Furthermore, sulfate availability plays a key role in early hydration processes and calcium silicate hydrate development, thereby influencing both rheological properties (Figure 6) [35].

Interestingly, the CS dihydrate group, despite its lower solubility, exhibited the shortest setting time (Figure 7) and the highest penetration resistance values (Figure 10) among tested groups, indicating that factors beyond dissolution kinetics contribute to setting behavior. This process can be explained by a nucleation (seeding) effect, where pre-existing CS dihydrate particles act as immediate crystallization nuclei [52,53,62]. Unlike CS hemihydrate and CS anhydrite, which require dissolution prior to precipitation, CS dihydrate provides ready-to-grow nucleation sites, enabling rapid crystal growth and the early formation of a rigid, interlocked structure. This seed-induced crystallization acceleration contributed to the reduced setting time (Figure 7), lowest washout volume (Figure 8), and shortest required time for reaching specific penetration resistance values (Table 3).

Therefore, the results shown in Figure 6 and Figure 7 demonstrate that setting behavior is controlled by solubility-driven supersaturation and nucleation site availability, providing an explanation for the observed balance between the flowability and setting time in CSC systems [14,46].

The method for measuring the setting time specified in ISO 6876 involves visually determining the hardening of the material, which has the drawback of relying on subjective judgment and can vary depending on the force manually applied by the operator. For materials with longer setting times, the testing process not only requires more time for the operator but also increases fatigue. The advantage of the new setting time measurement method, which utilizes a push–pull gauge device and the LabView program, is that it involves automated mechanical operation, requiring less skill and resulting in minimal variation between operators.

The washout resistance test method adopted for this experiment was inspired by the jet impingement method [63,64]. The jet impingement method was originally developed to assess differences in cell adhesion strength on biomaterials. It involves the controlled spraying of fluid from a nozzle tip into a temperature-controlled chamber, where it collides with a cell layer to determine the degree of cell attachment [63,64,65]. Similarly, this approach was employed to evaluate the washout resistance. The washed-out portions of the specimens were captured using a 3D scanner, and the volume was calculated using the Computer-Aided Three-Dimensional Interactive Application version 5 (Catia V5) program, enabling 3D modeling and calculations. This method offers a quantitative, objective, and reproducible way to measure the washout resistance.

The washout resistance results showed that the CS dihydrate addition group exhibited the lowest washout volume (Figure 8a,b), indicating the highest washout resistance. In contrast, the CS hemihydrate addition group, which had the highest flowability, demonstrated the lowest washout resistance (Figure 6 and Figure 8a,b).

Compressive strength is an important requirement for dental materials to resist occlusal stresses [40,66]. Figure 9 shows that the compressive strength results increased over time in all experimental groups. Additionally, the CS hemihydrate and CS dihydrate addition groups exhibited a higher compressive strength at 1 and 3 days compared with the CSC control group and the CS anhydrite addition group.

The penetration resistance of the mixed CSC materials during hardening from a gel state was evaluated by the time required to reach a specific penetration resistance value (MPa). The experimental groups that reached the specific penetration resistance in a shorter time exhibited faster hardening (Table 3). Indeed, the CS dihydrate addition group, which had the shortest setting time (Figure 7), also reached the specific penetration resistance values more quickly (Table 3, Figure 10). This rapid setting and corresponding rapid increase in penetration resistance of the CSC containing calcium sulfate dihydrate would improve procedural efficiency in endodontic applications, such as perforation repair, pulp capping, and retrograde filling. The penetration resistance showed a trend consistent with the setting time results. The anhydrite group exhibited the longest setting time and the slowest increase in penetration resistance (Figure 7 and Figure 10; Table 3), making it more suitable for clinical applications requiring prolonged working time and sustained plasticity, such as endodontic sealing procedures.

In the MTT assay tested with undiluted 100% leachate, the CSC-containing experimental groups showed a cell viability lower than 70% compared with that of the control group (without CSC). However, when the leachate was diluted to 25% or 50%, the CV of the tested CSCs satisfied the ISO requirement of a CV above 70% (versus the control), suggesting that there would be no cytotoxicity of concern. For the 25% extract dilution groups, the CS-hemihydrate-containing CSC showed a significantly higher CV compared with the CSC control group (CS-non-containing) (*p* < 0.05) (Figure 11).

It should be noted that the 100% leachate condition does not necessarily represent the actual biological environment encountered clinically. CSC may be used in various clinical applications, including pulp capping, root perforation repair, and retrograde filling, each involving different fluid dilution conditions. In root perforation repair and retrograde filling procedures, the material is exposed to blood and interstitial fluids that can dilute the released ions and soluble components, whereas pulp capping is performed in a more confined environment where such dilution is expected to be less extensive [7,53,67]. Therefore, the biological responses observed at lower eluate concentrations may more closely reflect clinical environment, whereas those observed with the 100% eluate may represent the initial local exposure immediately after material placement. Nevertheless, the present in vitro extraction model cannot fully reproduce the complex physiological conditions encountered in different clinical environments, including tissue buffering capacity, fluid exchange, and cellular interactions. Consequently, caution should be exercised when extrapolating the present cytotoxicity findings to clinical outcomes, and further in vivo investigations are required to validate the biological behavior of the material under relevant clinical conditions.

## 5. Conclusions

The selective incorporation of CS with different hydration forms into CSC resulted in marked changes in the physical properties. CS hemihydrate improved the flowability, whereas CS dihydrate significantly shortened the setting time and reduced the washout volume. These findings indicate that the hydration state of CS is a key factor governing the handling, setting behavior, and manufacturing of CSC.

Therefore, careful selection of the CS hydration form is essential for tailoring CSC properties to specific clinical applications and achieving an optimal material performance.

## Figures and Tables

**Table 1:** Relative proportions (wt%) of various crystalline phases of three calcium silicate cement clinker powder samples (CSC #1, CSC #2, and CSC #3), calculated using XRD-Rietveld analysis.

Crystalline Phase	Alite	Belite		Aluminate	Quartz	Cristobalite	Lime	Calcite	Grossular	Gehlenite	Etc.	Total
	C_3_S	β-C_2_S	γ-C_2_S	C_3_A	SiO_2_	SiO_2_	CaO	CaCO_3_	3CaOSiO_2_Al_2_O_3_	Ca_2_Al(AlSiO_7_)		
**CSC #1**		22.3	53.4	18.6	0.9	0.8			2.1	1.5	0.4	100
**CSC #2**	53.4	16.8		27.9	0.2		1.3				0.4	100
**CSC #3**	67.4	10.3		13.8	0.2	0.8	6.8	0.7				100

**Table 2:** Compressive strength (MPa) of calcium silicate cement (CSC) specimens after different curing periods.

	Group	CSC Control *	Anhydrite	Hemihydrate	Dihydrate
Aging Time					
**1 day**		24.42 (1.14)	25.36 (1.41)	31.69 (1.18)	35.13 (2.08)
**3 days**		26.85 (1.90)	31.14 (2.20)	36.49 (1.60)	38.63 (3.12)
**7 days**		28.85 (1.23)	35.56 (2.51)	37.35 (3.19)	40.22 (2.24)

* Values are presented as mean (SD). *n* = 7 for each group.

**Table 3:** Average elapsed time (min) until the penetration resistance reached specific values of 50 and 60 MPa (unit: min, elapsed time) against indenter penetration, with *n* = 5 replicates for each group.

	Groups	CSC Control	Anhydrite	Hemihydrate	Dihydrate
Penetration Resistance (MPa)					
50 MPa		8.53 (1.58)	29.45 (6.67)	20.31 (3.12)	5.69 (1.48)
60 MPa		11.05 (1.23)	45.12 (10.15)	24.49 (3.48)	7.03 (1.38)

## Data Availability

The original contributions presented in this study are included in the article. Further inquiries can be directed to the corresponding author.
